# The Value of Age-Friendly Public Health Systems in the Age-Friendly Ecosystem

**DOI:** 10.3390/geriatrics8030063

**Published:** 2023-06-02

**Authors:** Megan Wolfe, J. Nadine Gracia

**Affiliations:** Trust for America’s Health, Washington, DC 20036, USA

**Keywords:** healthy aging, age-friendly, public health

## Abstract

The United States population is living longer and healthier than ever. This enables our communities—and our society—to continue to benefit from our knowledge, experience, and energy as we age. The public health system is foundational for increasing life expectancy, and now it has the opportunity to further support older adult health and well-being. Trust for America’s Health (TFAH), in partnership with The John A. Hartford Foundation, launched the age-friendly public health systems initiative in 2017 with the goal of raising awareness within the public health sector of its many potential roles in healthy aging. TFAH has worked with state and local departments of health to build capacity and expertise in older adult health and has provided guidance and technical assistance to expand this work across the U.S. TFAH now envisions a public health system that has healthy aging as a core function. This paper aims to describe why the public health sector should adopt healthy aging policies and practices, how this is being operationalized at the state and local levels, and the value of age-friendly public health systems within the age-friendly ecosystem.

## 1. Demographics of Aging Population in America

The story of the older adult population in the United States is an amazing one of longevity and quality of life. Over the past ten years, the number of adults in the United States aged 65 and older increased by more than 34%, in part due to advances in public health, health promotion, and disease prevention [1]. By 2060, it is expected that 98 million people, or about one-fourth of the U.S. population, will be 65 or older [2]. The number of older adults, people aged 85 and older will grow from six million to 20 million by 2060.

The story of the older adult population also provides an opportunity to take stock in whether governmental and non-governmental systems are adequately prepared to ensure the highest quality of life and health for all Americans. Such preparation must include consideration of effective multi-sector partnerships and collaborations across the age-friendly ecosystem.

This rise in the number and proportion of older adults is important, but only paints part of the picture, as the older adult population is also becoming more racially, ethnically, and culturally diverse. In 2018, 77% of older adults were non-Hispanic white, 9% were Black/African American, 8% were Latino, and 5% were Asian. By 2060, the percentage of non-Hispanic whites is expected to drop to 55%, while the proportion of other racial groups will increase, with 21% of the population being Latino, 13% being Black/African American, and 8% being Asian [3].

## 2. Public Health and Healthy Aging

**Figure 1:**
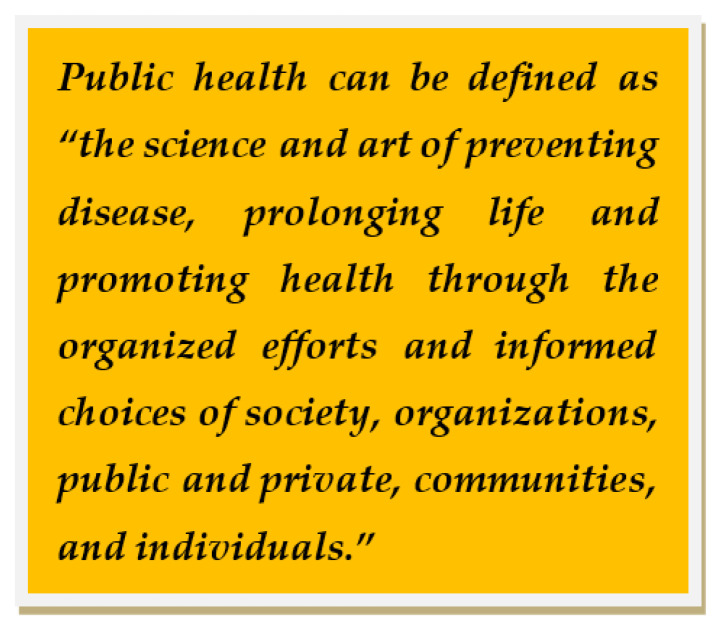
Introduction to Public Health. Available online at Introduction to Public Health|Public Health 101 Series|CDC (accessed on 20 March 2023).

The public health sector has contributed to advances in longevity over the last several decades. According to the U.S. Centers for Disease Control and Prevention (CDC), Americans’ life expectancy increased over the last 100 years by 62%, from 47.3 years in 1900 to 76.8 in 2000, with improvements in population health status at every stage of life [4]. Among the top public health achievements responsible for this significant advancement in longevity are tobacco cessation and control, identifying and immunizing against vaccine-preventable diseases, improving motor vehicle safety, and cardiovascular disease prevention. Nevertheless, these advancements have not been experienced across the population. One study found that the rate of individuals who smoke and who have high incomes in America dropped by 62% between 1960 and 2000, but the rate of Americans with low incomes who smoke dropped by only 9% [5]. Nor does the story of longevity in the U.S. apply equally across all races and ethnicities [6]. In 2019, the life expectancies of Black women and men were 78 and 71 respectively, compared to 81 and 76 for white women and men [7]. In addition, Alzheimer’s Disease and other dementias are twice as prevalent among Black individuals than among white individuals, and older Black people are more likely to suffer from heart disease than older white people. The structural drivers of these disparities include education level and literacy, as well as the conditions and environments in which the individuals were born, lived, worked, and aged—the social determinants of health. Notably, life expectancy in the U.S. dropped in 2020 and 2021, driven by the pandemic, but also attributable to drug overdoses and accidental injury [8]. However, life expectancy does not take into account productivity, independence, cognitive function, or any other measures of healthy aging. Many factors contribute to older adult health and well-being, and many of these factors are well within the domain of the public health sector. The data above underscore the need for public health that focuses not solely on a life course approach to health and well-being but also on addressing the underlying causes of health disparities. See the definition of public health in Figure 1.

## 3. Social Determinants of Health: Social Isolation and Caregiving Are Public Health Issues

For approximately the last 20 years, the public health sector has focused on developing expertise in the social determinants of health, which are defined by the CDC as “the nonmedical factors that influence health outcomes; the conditions in which people are born, grow, work, live, and age; and the wider set of forces and systems shaping the conditions of daily life. These forces and systems include economic policies and systems, development agendas, social norms, social policies, racism, climate change, and political systems” [9]. The social and economic issues that affect the health and well-being of older adults include social isolation and loneliness, access to affordable housing and transportation, availability of appropriate and quality caregiving services, access to nutritious food, and access to quality health care.

The University of Michigan’s 2020 National Poll on Healthy Aging revealed that the COVID-19 pandemic significantly increased feelings of loneliness (lack of companionship and isolation) among older adults [10]. A greater proportion of adults aged 50–80 felt a lack of companionship, felt socially isolated, and had infrequent contact with others outside of their homes during the early months of the pandemic than in 2018. In June 2020, more than half of older adults (56%) reported feeling isolated from others, compared to 27% in 2018. Such isolation had a severe impact on both physical and mental health. Social isolation is associated with negative health outcomes, especially for Black and Latino older adults. A new study from Johns Hopkins University found that social isolation increases the risk for developing dementia among older adults [11]. Among older adults, Black people, Latino people, and people with fewer years of education experience more loneliness, leading to higher levels of stress, poor cardiovascular health, depressive symptoms, and cognitive decline, among many other conditions [6]. Social isolation is now being recognized in the public health field as a serious public health risk and can be included in the broader definition of social determinants of health [12].

Caregiving is defined as providing assistance with another person’s daily needs. This assistance may include support with activities of daily living, such as bathing and dressing, help with meals, providing transportation, and financial assistance (for example, paying bills). A person is considered a caregiver whether paid or unpaid. Caregiving is also now considered an important public health issue for several reasons. Studies show that being a caregiver can be more harmful to older adults’ physical health than to their emotional health. Caregiving duties can curtail positive health behaviors, such as physical activity, adequate sleep, good nutrition, and seeking care for one’s own health concerns [13]. Moreover, caregiving can be physically taxing, requiring heavy lifting, navigating with wheelchairs or other assistive devices, and protecting against falls and other physical risks. As the older adult population grows, so will the need for both paid and unpaid caregivers. More than half of older adults aged 85–89 need a caregiver due to health conditions or functional limitations, and three out of four older adults under 90 require care through home health agencies or in institutional (long-term) settings [14]. The role of the public health sector in supporting caregivers can thus be viewed as a population health and prevention issue.

## 4. Life-Course Perspectives on Social, Economic, and Health Inequalities in Later Life

To fully appreciate the opportunities for public health engagement in and contributions to healthy aging, it is important to consider the impact that persistent inequities have on health across the life course. Cumulative aging recognizes that social and economic conditions that exist early in life, whether positive or negative, may be “accumulated” over the life course [6]. Recognizing that disparities experienced by older adults are exacerbated by early-life social and economic disadvantages can help to underscore the importance of public health prevention activities, from childhood to older age.

Prior to the COVID-19 pandemic, most public health departments did not prioritize healthy aging, in part due to the lack of a systematic approach to addressing older adult health and well-being, lack of funding and resources, and limited capacity and expertise. The aging services sector has historically had a leading role in addressing the needs of those living longer lives. Most public health programs that do exist to support older adult health needs are disease- and condition-specific, such as diabetes prevention, fall prevention, and addressing Alzheimer’s Disease and related dementias. Most federal and state policies designed to support older adult independence, such as Medicare, Medicaid, and the Older Americans Act, have not identified roles for public health, nor have they provided funding for public health departments to target services for those aged 65 and older.

## 5. What Is an Age-Friendly Public Health System?

Since 2017, Trust for America’s Health (TFAH) has partnered with The John A. Hartford Foundation (JAHF) to elevate healthy aging as a core public health function. This partnership resulted in the development of the age-friendly public health systems (AFPHS) initiative to expand the role of public health in improving older adult health. TFAH led a convening in 2017 to explore the potential roles in healthy aging, resulting in the Framework for Creating Age-Friendly Public Health Systems (the 5Cs Framework), which outlined five functional areas (referred to as the 5Cs Framework) in which public health could expand practice and programs to improve and support older adult health and well-being [15].

In 2022, TFAH revised the 5Cs Framework to include a new “C”—creating and leading policy, systems, and environmental changes to support and improve older adult health and well-being—in addition to changes to the other components, which were updated to reflect best practices and the need to address the social determinants of health as well as the well-being of caregivers. The Framework’s 6 Cs are:

1. Creating and leading policy, systems, and environmental changes to improve older adult health and well-being.
2. Connecting and convening multi-sector stakeholders to address the health and social needs of older adults through collective impact approaches focused on the social determinants of health.
3. Coordinating existing supports and services to help older adults, families, and caregivers navigate and access services and supports, avoid duplication, and promote an integrated system of care.
4. Collecting, analyzing, and translating relevant and robust data on older adults to identify the needs and assets of a community and inform the development of interventions through community-wide assessment.
5. Communicating important public health information to promote and support older adult health and well-being, including conducting and disseminating research findings and emerging and best practices to support healthy aging.
6. Complementing existing health promoting programs to ensure that they are adequately meeting the needs of older adults.

## 6. Exploring and Expanding Age-Friendly Public Health Systems

To first test the Framework, TFAH initiated a pilot project in Florida, with JAHF funding, working directly with 37 of Florida’s 67 county health departments. The 18-month pilot project resulted in the creation of new data systems to identify older adult health priorities, the establishment of new collaborations and partnerships to leverage existing programs and services, consideration of older adults in community health assessments and planning, and expansion of existing programs to include older adults. Significantly, almost all of the public health practitioners who participated in the pilot project noted that the increased awareness of the needs of older adults in their communities has had a profound impact on their planning and assessment processes. The success of the Florida pilot project led to national momentum, expansion of AFPHS into additional states, and public health coordination of multi-sector age-friendly efforts.

In the Florida communities where public health departments have become AFPHS, older adults were better served during the pandemic by existing multi-sector partnerships that were leveraged to identify older adults who needed at-home vaccinations, expand food deliveries, and address social isolation. From 2021–2022, TFAH facilitated a mentor–mentee project through which existing AFPHS county health departments coached peers in neighboring jurisdictions to adopt age-friendly practices. This project expanded the number of AFPHS county health departments in Florida to 50. County health departments have also included older adult priorities in their community health assessments and community health improvement plans, further sustaining healthy aging policies and programs.

TFAH was awarded a second round of grant funding to expand AFPHS, starting in 2019, to work intensively with two state departments of health to build age-friendly public health systems. Eleven states and one U.S. territory applied for the three-year grant funding, with Mississippi and Washington being selected for this opportunity.

Mississippi has a centralized public health structure with three public health regions. Regional Health Officers and Administrators provide oversight for clinics in 80 of Mississippi’s 82 counties. In each of the 82 counties, more than 16% of the total population is aged 65 and older. The Mississippi State Department of Health AFPHS project has benefited from strong and committed leadership within the department that is necessary to create and sustain policy and systems changes in governmental public health. The primary goals of the AFPHS work in Mississippi were to create an AFPHS 6 Cs action plan and to expand other age-friendly practices and partnerships. An AFPHS Advisory Committee was created to provide leadership support and external expertise, and an AFPHS Learning and Action Network was created to engage internal partners to advance planning and change. The Mississippi State Department of Health began implementation of the 6 Cs action plan in the spring of 2023, ensuring long-term sustainability of age-friendly programs and practices.

The AFPHS expansion in Washington was originally intended to follow the Florida model, with a Learning and Action Network created with teams of local health jurisdiction (local public health) and aging network teams. The COVID-19 pandemic postponed the recruitment process and shortened the time available for planning and implementation of AFPHS activities. However, five teams were created, and the Northwest Washington Indian Health Board (NWWIHB) joined the project in the last year of the grant. The county-based teams and the NWWIHB developed action plans aligned with the 6 Cs Framework, focusing on emergency preparedness, using data to inform strategic planning, expanding fall prevention partnerships, and working to understand and counter ageism. Engagement with the NWWIHB provided the first opportunity for AFPHS to be implemented in a culturally and linguistically appropriate manner within a tribal elder community. The partnership also elevated the need for tribal health assessment and the need for more robust demographic and health-related data on tribal elders.

TFAH also helped to facilitate the creation of older adult data profiles for both Mississippi and Washington. Through a connection made by TFAH, the Mississippi State Department of Health collaborated with the University of Massachusetts, which helped to develop the profiles for all 82 of the state’s counties. Similarly, TFAH connected the Washington State Department of Health with the consulting firm Altarum, which resulted in the development of older adult profiles for all 37 of Washington’s counties. These data profiles provide valuable information on the health and well-being of the older adults in each state, enabling more effective prioritization of healthy aging activities as well as the channeling of limited resources. For example, the Washington profiles include not only the demographic growth of residents aged 65 and over, but also the chronic conditions of those residents, enabling public health practitioners to better understand the populations they serve and target interventions and resources to prevent escalation of those conditions.

## 7. COVID-19, Older Adults, and the Public Health Workforce

The COVID-19 pandemic had a disproportionate impact on older adults, underscoring the need and value of public health attention to this growing population. According to CDC data, 80% of deaths attributable to COVID-19 were of individuals aged 65 and older, and most of those occurred in long-term care facilities [16]. Furthermore, Black and Latino older adults were two to three times more likely to be hospitalized or die if they become infected with SARS-CoV-2 [17].

The pandemic, which necessitated the implementation of mitigation strategies such as physical distancing, masks, and quarantine to protect vulnerable older adults from COVID-19 infection, significantly exacerbated negative impacts of social isolation with the closure of eldercare facilities to outside visitors, effectively separating residents from their loved ones [18]. In addition, the pandemic caused fear, financial worry, depression, and other mental health concerns. The impacts of physical distancing and facemask requirements on older adults living with dementia are far worse, as these individuals rely upon routine and familiar faces to function.

Public health departments at the federal, state, local, territorial, and tribal levels have played a vital role in the COVID-19 pandemic response, working tirelessly at the frontlines to protect the public’s health. However, the pandemic exposed significant gaps in the nation’s public health infrastructure, challenging the public health response due to chronic underfunding. In 2021, the U.S. spent US $4.3 trillion on health (including 28% on private insurance, 21% on Medicare, 17% on Medicaid, 10% on out-of-pocket expenses), but only 3–5 percent of those health expenditures targeted prevention and public health [19].

The pandemic also underscored the need to bolster the health and public health workforce. Shortages existed even before the public health emergency was declared. In 2008, the Association of Schools and Programs in Public Health warned that by 2020, “the nation will be facing a shortfall of more than 250,000 public health workers” [20]. An October 2021 analysis conducted by the de Beaumont Foundation and the Public Health National Center for Innovations found that state and local public health departments need an 80 percent increase in workforce size to ensure basic public health services to the nation [21]. As the U.S. population continues to age, this workforce will need to build expertise in aging issues, develop more robust data systems to identify older adult health needs, and engage in strong multi-sector partnerships to leverage limited resources.

## 8. Building an Age-Friendly Ecosystem

A key strategy for tackling these issues is the development of local, state, and national age-friendly partnerships, or what is coming to be known as the age-friendly ecosystem. The age-friendly ecosystem can be defined as a comprehensive, collectively built, ever-expanding platform whose goal is to improve the quality of life for older adults through enhanced collaborative impact. All of the age-friendly models—age-friendly public health systems, age-friendly health systems, age-friendly communities, age-friendly universities, and others—have strengths and unique contributions for healthy aging. TFAH is working with JAHF and other organizations to identify and implement strategies for aligning these systems and sectors. For example, some local health departments are using aligned assessments to prioritize older adult health and social needs. State and local health departments can collaborate with health care providers to enhance care coordination for older adults. Public health practitioners can work with community-based aging services providers to ensure that nutritious meals are reaching all who need them.

Many state and local health departments are leading these coordinated efforts and TFAH has developed an age-friendly public health systems recognition program to honor this leadership and promote sustainable healthy aging policies. The recognition program is based on the AFPHS 6 Cs Framework, requiring development of action plans that include public health activities in each of the 6 Cs. Three state departments of health (California, Mississippi, and New York) and 20 local health departments have achieved recognition. The recognition program also acknowledges individuals who are committed to building their knowledge and expertise in age-friendly public health approaches through the AFPHS champion recognition initiative. Sixty individuals across nine states have been designated as AFPHS champions, representing public health, aging services, and academia.

## 9. Conclusions and Next Steps

Existing age-friendly initiatives include not only age-friendly public health systems, but also age-friendly health systems, age-friendly communities, dementia-friendly communities, and multi-sector plans for aging. Although these movements are often siloed and may lack a shared vocabulary, common measures, and shared goals, TFAH and JAHF are exploring coordinated activities to achieve collective impact. Mutually beneficial partnerships among age-friendly leaders can foster the creation of an age-friendly ecosystem, providing the momentum and coordination to strengthen the intended age-friendly impact. Adoption of age-friendly public health practices and policies is essential for older adult health and well-being in emergency and non-emergency times. The public health sector’s experiences in ensuring an appropriate and robust response to the health and social needs of older adults during the COVID-19 pandemic provided a strong example of the roles that public health departments can play to advance healthy aging. Becoming an age-friendly public health system is a crucial advancement to demonstrate commitment to the health of all people across the life course.

## Data Availability

Not applicable.
